# Implications of the Cass Review for health policy governing gender medicine for Australian minors

**DOI:** 10.1177/10398562241276335

**Published:** 2024-08-31

**Authors:** Alison Clayton, Andrew James Amos, Jillian Spencer, Patrick Clarke

**Affiliations:** School of Historical and Philosophical Studies, 153396 University of Melbourne, Melbourne, VIC, Australia; Division of Tropical Health and Medicine, College of Medicine and Dentistry, 104397 James Cook University, Townsville, QLD, Australia; Greater Brisbane Clinical school, University of Queensland, Brisbane, QLD, Australia.; Faculty of Health and Medical Science, University of Adelaide, Adelaide, SA, Australia

**Keywords:** gender dysphoria, gender-affirming treatment, youth mental health care, administrative psychiatry, public health

## Abstract

**Objective:**

To summarize the key recommendations of England’s independent inquiry into gender identity services for children and young people (the Cass Review) and to evaluate their relevance to Australian health policy.

**Conclusions:**

The Cass Review’s findings and recommendations have clear applicability to Australian health policy. As a matter of priority, Australian health authorities need to seriously engage with the Cass Review's findings and recommendations. To not do so will put the health and well-being of vulnerable children and young people at risk.

Over the last decade, in Australia, as in many countries, there has been a sharp rise in the numbers of youth experiencing gender-related distress and/or identifying as transgender and presenting to youth gender clinics.^1^ Previously, referrals were mostly biological sex males, whereas the current cohort is dominated by adolescent biological sex females, and many have neurodevelopmental and co-existing psychiatric disorders.^1,2^ Australian youth gender clinics utilize a multidisciplinary approach and provide gender-affirming psychosocial and medical treatments tailored to developmental stage and individual needs (see Box 1).^1,3^ Henceforth, we will use the umbrella term the gender-affirming care treatment model (GACTM) to describe this clinical approach.

The GACTM for youth is controversial. As recently summarized, the research on the treatment benefits and harms of key GACTM interventions (puberty blockers [PBs] and cross-sex hormones [CSHs]) for youth with gender dysphoria/gender incongruence (GD/GI) ‘has significant conceptual and methodological flaws, ... the evidence for the benefits of these treatments is very limited, and ... adequate and meaningful long-term follow-up studies are lacking’.^2^

In 2020, NHS England, in response to the changing referral patterns and concerns that the GACTM was harming minors because of fundamental problems with the model as well as poor governance of its implementation, commissioned an independent review – the Cass Review.^4^ The final report was released in April 2024. Its findings are critical of key aspects of the GACTM and the incremental ‘creep of unproven approaches into clinical practice’ without appropriate monitoring, oversight and regulation (p. 231).^4^

The GACTM used to treat minors with GD/GI in Australia’s public youth gender services is similar to the one criticized by Cass.^5^ Furthermore, a systematic review, commissioned by Cass, evaluated 23 international and national guidelines/clinical guidance publications.^6^ This found that the Australian guidelines,^3^ which define the GACTM for youth in Australia, lack methodological rigour and editorial independence, and they were not recommended for use in practice.^4,6^

To date, Australian medical authorities have largely ignored the Cass report’s findings and recommendations. Claims have been made that the Cass Review’s methodology is flawed and that Australian practice already conforms to Cass’s recommended practice.^7^ Given Cass’s critique of the Australian guidelines and recommendation for the NHS to establish a PB clinical trial, whereas in Australia PB remain an integral part of the GACTM,^3^ this latter claim is implausible.

In this article, to promote the mental health, social welfare, and physical well-being of gender-distressed Australian minors and their families, we summarize the Cass Review’s recommendations that are most relevant to Australian practice. We then respond to some of the arguments being made by Australian gender clinicians to justify ignoring them. The Australian Professional Association for Trans Health (AusPATH) describes itself as Australia’s peak body for professionals involved in the health, rights, and well-being of all gender-diverse people. To our knowledge, all the clinicians leading Australia’s child and adolescent gender clinics are aligned with AusPATH. The Vice President of AusPATH has made several public statements regarding the Cass Review.^7^ These statements provide a useful template to critically analyse the claims that the final report of the Cass Review is methodologically flawed and irrelevant to the Australian setting. In our response to these statements, we interweave responses to other published criticisms of the Cass Review.^8–11^ Our brief paper does not purport to cover the totality of the debate about these complex issues but does aim to enhance and stimulate the level of debate about the Cass Review and its implications for Australian health policy.

## Analysis and evidence

### The Cass Review

England’s^4^ independent review of gender identity services for children and young people was conducted over a 4-year period and is based on commissioned systematic reviews of the evidence (see Table 1) alongside engagement with key stakeholders. While some of its key recommendations are specific to the local English setting, many are of international importance. In particular, the review describes the weak (described as ‘threadbare’ by BMJ editor-in-chief^12^) evidence base for some of the key interventions of the GACTM for gender dysphoric youth, namely, social transition, PB, and CSH.

**Box 1.** Gender-affirming psychosocial and medical interventions for under 18-year-olds described in the Australian Guidelines^3^ include

• Gender-affirming psychosocial support (e.g. use of preferred name and pronouns; assistance with understanding of gender identity; assessment/treatment/referral for mental health issues/complex psychosocial issues; advocacy for a gender-affirming home/school environment for the child)
• Voice and communication training
• Fertility counselling and preservation
• Social transition, which may involve adoption of gender-affirming hairstyles, clothing, names, and pronouns
• Puberty blockers to prevent undesired physical changes during puberty
• Cross-sex hormones to promote desired physical changes
• Referral for chest reconstructive surgery for eligible transmasculine minors.

**Table 1.** The Cass Review’s commissioned independent evidence review and research program from the University of York.^4^

A. University of York: The epidemiology, care pathways, outcomes, and experiences of children and adolescents experiencing gender dysphoria/incongruence: a series of linked systematic reviews and an international survey:
(1) Impact of social transition in relation to gender for children and adolescents: a systematic review.
(2) Gender services for children and adolescents across the EU-15+ countries: an online survey.
(3) Psychosocial support interventions for children and adolescents experiencing gender dysphoria or incongruence: a systematic review.
(4) Clinical guidelines for children and adolescents experiencing gender dysphoria or incongruence: a systematic review of guideline quality (Part 1).
(5) Clinical guidelines for children and adolescents experiencing gender dysphoria or incongruence: a systematic review of recommendations (Part 2).
(6) Interventions to suppress puberty in adolescents experiencing gender dysphoria or incongruence: a systematic review.
(7) Masculinizing and feminizing hormone interventions for adolescents experiencing gender dysphoria or incongruence: a systematic review.
(8) Characteristics of children and adolescents referred to specialist gender services: a systematic review.
(9) Care pathways of children and adolescents referred to specialist gender services: systematic review.
(The published journal articles are open access and available at https://adc.bmj.com/pages/gender-identity-service-series)
B. University of York, Qualitative Research Program:
Young People Distressed by Gender Dysphoria: A Qualitative Study Exploring the Perspectives of Young People, Parents/Carers and Care Professionals.

The main thrust of the Cass Review’s recommendations is to move the management of gender dysphoric youth away from the GACTM towards a focus more in keeping with standard psychological and psychotherapeutic approaches provided by local services with a broad range of skills in child and adolescent mental health (see Box 2). Medical intervention will only be available through referral to tertiary centres, and every case considered for medical treatment will be discussed by an independent national multi-disciplinary team. The review found there was insufficient evidence to support the use of PBs as routine clinical treatment. Stand-alone use of PBs for the indication of GD/GI will now only be available from the NHS under an ethics committee-approved research protocol. The review recommends extreme caution in the use of CSH, only available to over 16-year-olds, and the NHS has yet to announce the conditions of their use. Similarly, the review discourages early childhood full social transition, noting the lack of evidence for benefits and the risks this intervention might pose. All interventions, including psychological approaches, require better evidence and Cass advises to setting up a rigorous research program to improve the evidence base. It is also important to highlight that the Cass Review does not just focus on interventions but challenges many key assumptions, such as the aetiology and natural history of youth gender dysphoria, that underpin the GACTM of youth gender dysphoria.

**Box 2.** Key Differences Between the Cass Review Recommendations^4^ and Australian Guidelines^3^ on Gender-Affirming Care Treatments

• Cass does not stipulate a gender-affirming care treatment model; the Australian guidelines are underpinned by the gender-affirming care treatment model.
• Cass recommends a cautious approach to social transition, especially full social transition for pre-pubertal children, and notes the lack of evidence for psychological benefit; Australian guidelines recommend that social transition decisions should be child-led and that evidence suggests it is of psychological benefit.
• Cass recommends an increased focus on the development of standard evidence-based psychological approaches as a primary treatment modality for gender-related distress; Australian Guidelines give minimal attention to psychological approaches as a treatment modality for GD/GI.
• Cass/NHS: PBs will only be available as part of a human research ethics committee-approved clinical trial; Australian guidelines: PBs remain a routine treatment option.
• Cass: CSHs are currently available for over 16-year-old, but extreme caution is recommended and tertiary centre referral is required (the NHS is yet to provide specifics); Australian guidelines: do not stipulate a lower age limit for CSH and they can be commenced in primary care settings if certain criteria are met.
• Cass: does not consider surgery, as this is not part of NHS treatment options for minors; Australian guidelines: chest reconstructive surgery is an option for transmasculine minors.
• Cass: the decision for any under 18-year-old commencing a medical pathway will need to be discussed at a national multidisciplinary team independent of the young person’s treating clinician. Australian guidelines: the treating team can authorize commencement of medical treatment if specific criteria are met (e.g. diagnosis of GD made by a mental health clinician, and if desired/consented to by young person, and if parents/guardians agree/consent).
• Cass: Detransition and detransitioners’ clinical care needs discussed; Australian guidelines: no mention of detransition/detransitioners.

### Criticisms of the Cass Review

#### The Cass Review recommendations are at odds with the current evidence base, expert consensus, and most clinical guidelines around the world

The Cass Review’s recommendations are not at odds with the evidence base. To the contrary, they have been informed by eight independent systematic reviews of the evidence that were commissioned by Cass.^4^ These have been published as journal articles in the Archives of Disease in Childhood to coincide with the release of the Cass Review (see Table 1). The findings of the commissioned reviews on PBs and CSH are in keeping with numerous other systematic reviews, which largely concur that there is a lack of reliable evidence for their benefits to mental health and quality of life in youth with GD/GI.^2,13^ The World Health Organization (WHO) has acknowledged this in a recent statement, noting that the evidence base for commencing medical gender reassignment interventions during developmental years is ‘limited and variable’, and therefore the planned WHO guidelines on the health of trans and gender-diverse people will not pertain to children and adolescents.^14^

Cass’s recommendations are at odds with expert medical opinion that supports the GACTM for minors. However, they are in line with a substantial body of expert medical opinion that does not support the GACTM for minors.^15–17^ There is also no expert consensus regarding many aspects of child and adolescent GD/GI – diagnostic criteria are controversial and lack predictive validity; aetiology is debated; the meaning of changing demographics is disputed; the natural history is uncertain; the most appropriate treatment approach and the appropriate aims of treatment contested; and the capacity of adolescents to consent to medical and surgical treatments called into question.^18–20^

Criticisms of the Cass Review predating the final report complained about the review’s lack of engagement with stakeholders, international evidence, other consensus-based guidelines, international experts in youth gender dysphoria, and reliance on what were claimed to be flawed NICE systematic reviews.^10,11^ However, these criticisms lack relevance to the final report, which clearly describes Cass’s engagement with a wide range of community stakeholders, youth gender experts, international evidence, international guidelines, and a reliance on newly commissioned and up-to-date systematic reviews.^4^

We agree that some of Cass’s recommendations are at odds with some guidelines. However, they have similarity to others, for example, the Swedish and Finnish guidelines.^5^ Further, the systematic review, commissioned by Cass, of the available clinical guidelines for youth with GD/GI found that, apart from Sweden and Finland, other guidelines, including Australia’s, lacked methodological rigour – for example, by not systematically reviewing the evidence nor making clear links between the evidence and recommendations, and were not recommended for use in practice. Most were heavily reliant on two guidelines – the World Professional Association for Transgender Health (WPATH) and Endocrine Society, which themselves lack developmental rigour and were linked through cosponsorship.^6^

WPATH, in its critique of Cass, claimed that its 2022 ‘Standards of Care’ (SOC8) ^22^ were based on more systematic reviews than Cass.^8^ However, to our knowledge only two of the WPATH commissioned systematic reviews have been published. This means there is a concerning lack of transparency regarding the findings of the other WPATH reviews. Furthermore, WPATH SOC8’s adolescent chapter states that ‘a systematic review regarding outcomes of treatment in adolescents is not possible’ and a ‘short narrative review is provided instead’ (p.46).^21^ This is a puzzling statement, given the numerous published systematic reviews on PBs and CSH treatments for adolescents. In any event, this failure to use systematic reviews in its evaluation of the evidence for its management recommendations of GD/GI in adolescence substantially undermines the credibility of WPATH’s SOC8 and its claims to superiority in its official response to Cass’s final report.

#### In Australia, guidelines for gender-affirming care for young people are already holistic, individualized, and include the involvement of multidisciplinary teams of clinicians with all kinds of areas of expertise, to help and support young people in their gender journey

In Australia, it is under the umbrella of the GACTM that the so described ‘holistic’, ‘multidisciplinary’, and ‘individualized’ assessments are undertaken. In contrast, the Cass Review does not endorse the GACTM. This difference is of key importance when considering the meaning of ‘holistic’, ‘multidisciplinary’, and ‘individualized’.

The GACTM views gender diversity as a normal variant of human development and gender identity as an innermost concept of self as male, female, or other.^3^ It posits that elevated rates of depression, anxiety, self-harm, and suicide in gender-diverse children and adolescents are primarily due to experiences of stigma, discrimination, social exclusion, bullying, and/or barriers to gender-affirming care. Thus, the emphasis is on other psychiatric conditions or psychosocial circumstances as secondary, or coincidental, to GD/GI; rather than as possibly primary and causal to a GD/GI presentation.^3,18,21^ These co-existing conditions might need referral for separate treatment but there is also an expectation that psychiatric conditions, for example, eating disorders, can resolve with GACT and that they should not necessarily impede key interventions such as social and medical transition.^3,21^ Under this GACTM, it appears that the multidisciplinary gender clinic team largely functions to ensure all aspects of gender-affirming treatment, if desired by the eligible child/adolescent, are supported. The requirement to ensure any other coexisting medical or psychosocial conditions are being appropriately attended to is just part of any standard clinical care approach.

In contrast, Cass notes that alongside biological factors, psychosocial circumstances (such as trauma, homophobia, social influence) and mental health conditions might contribute to the development of youth GD/GI, rather than just being secondary or coincidental.^4^ Under the Cass model, the multidisciplinary assessment is geared towards identifying elements in these various domains relevant to the individual patient’s GD/GI. Cass notes the critical importance of a formulation to inform an individualized management approach which is developed by a collaborative process considering patient values, clinical expertise, and research evidence.^4^ In this model, GD/GI may well resolve with maturity, treatment of any co-existing psychiatric conditions, and/or supportive psychosocial care or psychotherapy – such as trauma-informed therapy or family therapy as indicated for each individual case. Importantly, this type of therapy does not aim to ‘change someone’s identity’ but validates a young person’s experience while opening space for self-reflection about their experiences and help with alleviating distress. This is not conversion therapy.^4,22^

A further important issue to note is the difference between rhetoric versus practice. Just like Australian gender clinics, the Tavistock youth gender clinic also claimed to be undertaking holistic, individualised, and multidisciplinary assessments.^23^ It was only under review that it was found that practice was not living up to the rhetoric.^4^ In our professional practice, we are aware of parents and ex-patients who report unsatisfactory experiences of assessment at Australian gender clinics, including, for example, failure to identify and manage conditions such as trauma and autism. These reports suggest that practice in Australia’s gender services may share the problems reported at the Tavistock, including that the focus on gender-related issues may overshadow other issues negatively impacting on a young person’s well-being. An inquiry which includes an independent audit of gender clinic files may be the only way to determine if the same issues that beset the Tavistock clinic are occurring in Australia.

#### Applying the findings and recommendations of the Cass Review to the care of young people in Australia is fundamentally flawed because it looked specifically at the NHS system; whereas the way that gender-affirming care is accessed and provided in Australia is substantially different

As already discussed, this is incorrect. Some of Cass’s recommendations apply specifically to the NHS system but many are of international importance. For example, the evidence base for the benefits of puberty blockers is not higher in Australia than in England. The possibility that a young person’s GD/GI may be secondary to other mental health or psychosocial issues is not different for children in Australia than in England.

#### There are many areas of medicine, especially paediatrics, where it is not feasible or ethical to conduct randomized control trials to collect the ‘highest quality’ of evidence

This issue is substantially more complex than this statement suggests. There have been numerous randomized controlled trials (RCTs) undertaken in paediatric medicine, although more needs to be done to encourage this.^24^ If there is substantial uncertainty about whether a treatment will benefit patients, then RCTs are ethical. One area of note is paediatric oncology. The history of paediatric oncology demonstrates that enrolling children and adolescents with rare and life-threatening conditions in high-quality randomized trials is not only possible but also improves outcomes. Furthermore, in response to criticism regarding the ethics of withholding new treatments from the patients randomized to the standard treatment arm of the study, research has shown that new treatments tested in randomized controlled trials are, on average, as likely to be inferior as they are to be superior to standard treatments.^25^

In addition, although not optimal, there are some areas of paediatric medicine where interventions are informed by RCTs undertaken in adults with the same conditions and which have shown good evidence of benefit. However, adult gender medicine is characterized by a lack of RCTs, and the evidence base for the claimed mental health benefits of GAT in adults is weak.^13,18,26^ In some clinical situations, where RCTs are not possible, then high-quality longitudinal observational studies may provide acceptable evidence but should always be subject to ongoing review.^24^ In youth gender medicine, it is not only the lack of RCTs that is the issue but it also the dearth of high-quality longitudinal observational studies. Of note, Cass does not stipulate that clinical trials need to be RCTs. Cass recommends that more evidence is required on psychological treatment approaches, as well as medical interventions, and that the National Institute for Health and Care Research commission a living systematic review to inform the evolving clinical approach, to ensure that services are operating to the highest standards of evidence.^4^

#### When you have multiple observational studies looking at a particular intervention and those studies are producing similar findings, the cumulative evidence becomes compelling

When all studies are flawed by similar limitations (e.g. small samples, non-responder bias, attrition bias, placebo effects, and unmeasured confounding) and/or when systemic biases are operative (e.g. measurement, publication, and reporting bias) and/or when effect sizes are small and inconsistent, then the evidence is not compelling. This is the situation in youth gender medicine.^4,13,18,26,27^ There are multiple examples in the history of medicine where there has been medical consensus for an intervention and/or observational studies have indicated the effectiveness of an intervention but later this is contradicted by more rigorous studies. A common pattern is that although there is a weak evidence base, a practice gains premature acceptance largely through vocal support from prominent advocates who have faith in it. Later, it is recognized as not being as beneficial as claimed and as causing more harm than acknowledged, but removing the contradicted practice often proves difficult.^28–30^ While it is acknowledged that this is an issue for other fields of medicine, this does not mean that the weak evidence base does not matter for youth with GD/GI. We would argue that when the treatment being considered is an invasive intervention pathway which holds risks of serious harms (including to fertility and sexual function) for a poorly understood condition in children and adolescents, then, more caution is needed and there is a greater onus on the clinicians promoting such treatments to provide rigorous evidence for the benefits and to show that there are no less risky treatment alternatives.

## Discussion: Implications for health policy in Australia

The strongest Australian advocates of the GACTM for youth acknowledge that the evidence base for its practices is so weak that it is primarily based on clinician consensus.^3^ In addition, the Cass Review specifically identified weaknesses of methodological rigour and editorial independence in the Australian guidelines, which underpin the clinical approach of all public youth gender services in Australia, and did not recommend them for use in practice.^4–6^ In this context it is imperative that Australian medical authorities engage, as a matter of priority, with the comprehensive and rigorous evidence and authoritative advice provided by Cass. Box 2 lists some of Cass’s key recommendations and demonstrates key differences with Australian youth gender medicine practice. We use the example of puberty blockers to illustrate some general points.

The Cass Review unequivocally condemned the introduction and rapid expansion of puberty blockers in England as poor medical practice.^4^ Despite their own ‘early intervention’ PB study not demonstrating ‘improvement in psychological well-being’, in 2014 the English youth gender clinic moved PBs from being available as a research-only option to being a routine treatment option available to a rapidly expanding group of patients with very different characteristics than stipulated in either the Dutch or UK PB studies (p.70-73).^4^ Little effort was made to systematically and rigorously evaluate whether such treatment was improving patient health or causing significant side effects. Cass concluded that ‘the adoption of a medical treatment with uncertain risks, based on an unpublished trial that did not demonstrate clear benefit, is a departure from normal clinical practice’ (p. 73).^4^ This suggests, as recently argued in a paper in this journal, that political pressure to increase access to rights may have overcome the usual medical safeguards designed to maintain patient safety.^31^

In widely implementing an inadequately tested treatment, PBs, for youth with GD/GI, Australian practice paralleled that of England. Offering treatments based on inadequate evidence, and without adequate knowledge of benefits and risks, is unethical.^12^ In addition, Cass has raised concerns about systemic weaknesses with the governance of innovative clinical practice in England, which allowed for the ‘creep of unproven approaches into clinical practice’ and an excessive permissiveness with ‘off-label’ prescribing (pp. 74, 231).^4^ The situation seems similar in Australia, and questions have previously been raised about whether NHMRC guidance regarding the importance of distinguishing between experimental treatments, which should only be introduced under a formal research protocol, and innovative clinical practice have been followed.^13^

In our opinion, Australian health authorities should, as a matter of urgency, institute an independent investigation to review the practices in Australian youth gender clinics and consider stipulating that puberty blockers for GD/GI should only be prescribed under ethics committee-approved clinical trials. The role played by non-medical political pressure groups pushing for changes to practice and the ethical and legal responsibilities of the authors of medical guidelines that misinform also need to be investigated.

## Conclusions

To date, the overwhelming response of Australian youth gender clinicians, medical colleges, and health authorities to the Cass Review has been one of silence and dismissal rather than serious engagement. This is of grave concern. The Cass Review, like recent changes in health policy direction for youth with GD/GI in several European countries, has clear applicability to Australia. It represents an opportunity for clinical leadership within Australia to demonstrate a prudent and evidence-based approach towards the issue of the model of care provided to these vulnerable children. This would serve the best interests of Australian youth struggling with gender distress, and their parents and carers. To further this aim, we recommend that a rigorous, independent, and transparent inquiry into Australian youth gender clinics and the role of medical authorities, including medical colleges, as well as non-medical political pressure groups, be undertaken as a matter of urgency.
